# 

**Published:** 1861

**Authors:** 


### CHICAGO MEDICAL JOURNAL.

**Terms, $2.00 per Annum.**

### CONTENTS OF OCTOBER & NOVEMBER NOS.

ORIGINAL COMMUNICATIONS.

PAGE.

Introductory Address—Rush Medical College, &c. By De Laskie Miller, M. D., Prof. of Obstetrics and Diseases of Women and Children.... 563

Cases of Placenta Prævia. By Jonathan W. Brooks, M. D., of Chicago... 586

SELECTED.

Spina Bifida, Treated by Iodine—Cure by one Injection. By Daniel Brainard, M. D., Prof. of Surgery in Rush Med. Col., etc............... 597

Clinical Lecture on Cerebral Symptoms Independent of Cerebral Disease. Delivered at the Hospital for Sick Children, Jan. 26, 1861, by Chas. West, M. D., Physician to the Hospital........................ 601

New Anæsthetics—Theory of their Action. By Chas. T. Jackson, M. D.. 613

New Surgical Principles........................................ 616

Notes on the Narcotics. By Edward Parrish, Lecturer on Pharmacy, Philadelphia............................................ 617

Lecture on Dentition and its Derangements. By A. Jacobi, M. D., Prof. of Infantile Pathology and Therapeutics........................ 622

On the Molecular Theory of Organization. By J. Hughes Bennett, M. D., F. R. S. E., Prof. of the Institutes of Med., and Senior Prof. of Clinical Med. in the Univ of Edinburgh........................ 629

On the Mode of Action of Alcohol in the Treatment of Disease. By Edw. Smith, M. D., L. L B., F R. S., Asst. Physician to the Hospital for Consumption and Diseases of the Chest, Brompton, London, 639

Treatment of Uterine Inflammation by Nitrate of Silver and other Substitutive Agents. By E. J. Tilt, M. D., M. R. C. P ... ............. 660

Convenient Formulæ........................................ 669

Lead in the Treatment of Phthisis........................... 672

EDITORIAL.

Editorial and Miscellaneous........................................ 676

